# Supplementary material for: Non-native ants are breaking down biogeographic boundaries and homogenizing community assemblages
Source: Nat Commun. 2024 Mar 13;15:2266. doi: 10.1038/s41467-024-46359-9 (PMC10937723; doi:10.1038/s41467-024-46359-9)
Supplement: Supplementary file 1 — Supplementary Information [file 41467_2024_46359_MOESM1_ESM.pdf]

Supplementary information for

Non-native ants are breaking down biogeographic boundaries and homogenizing community assemblages.

Lucie Aulus – Giacosa[1*], **Sébastien Ollier**[1,2], **Cleo Bertelsmeier**[1*]

[1] Department of Ecology and Evolution, Biophore, UNIL – Sorge, University of Lausanne, 1015, Lausanne, Switzerland

[2] Université Paris – Saclay, CNRS, AgroParisTech, Ecologie Systématique Evolution, 91405, Orsay, France

Corresponding authors: lucie.aulus@unil.ch or cleo.bertelsmeier@unil.ch

The pdf file includes:

Supplementary Figures 1 to 6.

Supplementary Methods.

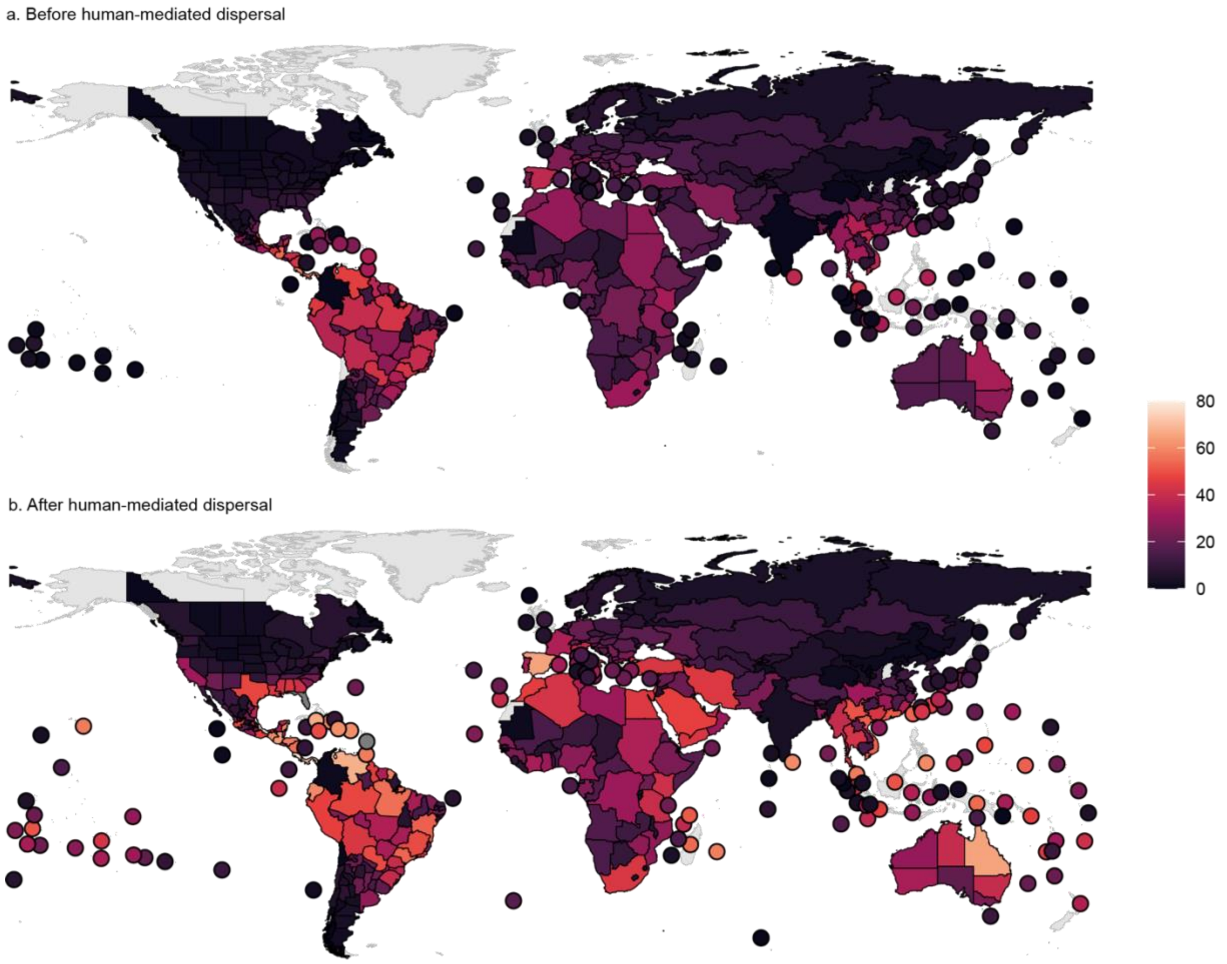

Supplementary Figure 1. Non-native ant species richness by polygon (a) in their native range (before human-mediated dispersal) and (b) in their current range (native + non-native ranges after human-mediated dispersal).

Species richness varies from 1 to 60 species in their native range (a) and from 1 to 85 species in their current range (b). Grey polygons represent subcountry political regions where non-native ant species are not described yet. The current range (b) is equivalent to the sum of the native and the non-native ranges. Source data are provided as a Source Data file.

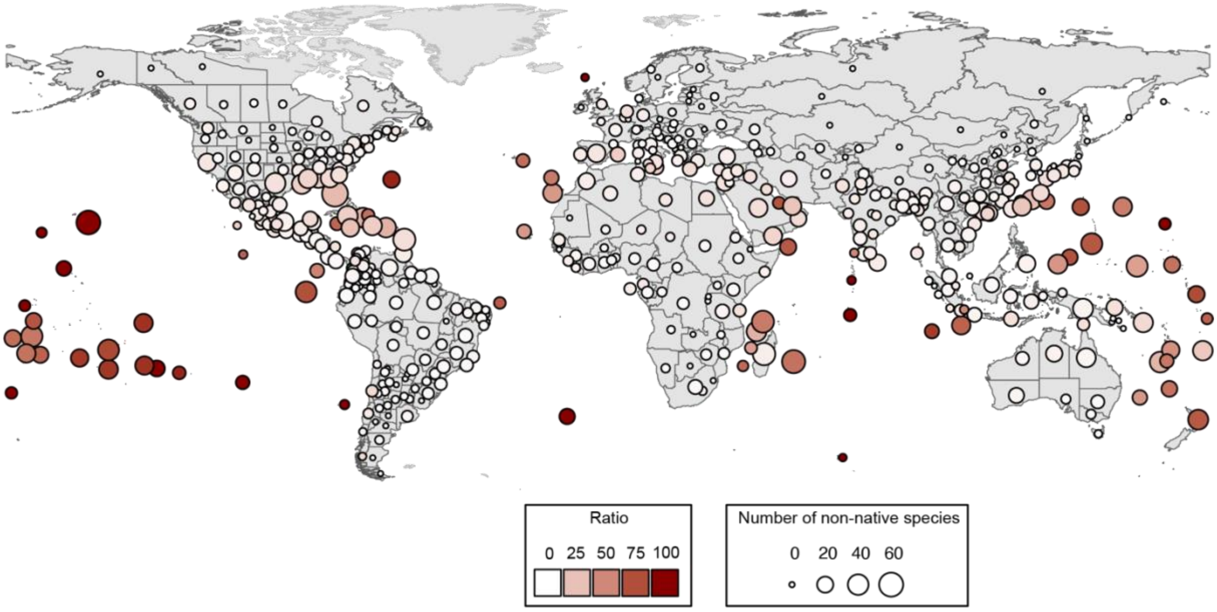

Supplementary Figure 2. Ratio of non-native ant species compared to all native ant biodiversity in each polygon and number of non-native ant species introduced by polygon. Ratio value are displayed in color, from white (very few non-native species in the communities) to dark red (high proportion of non-native species in the communities). The size of the circle displays the number of non-native species that has been introduced in each considered polygons (536 polygons with ant species on 546 polygons). Source data are provided as a Source Data file.

Supplementary Methods: Sensitivity analysis of the delineation of bioregions before human-mediated dispersal: ***Testing if non-native ant species are random species pool of all native ant diversity.***

As compositional dissimilarity (β_{sim}) between polygons is sensitive to species richness, one may expect that historical bioregions (species in their native range) of non-native ant species differ from all ant species. To explore the sensitivity of the delineation of bioregions to species richness (species pool considered), we generated 7 species pools, by selecting randomly ten times groups of 300, 400, 500, 1000, 2000, 5000, and 10,000 species among all ant species, generating in total 70 presence-absence matrices. For each of those 70 presence-absence matrices, we calculated pairwise matrices of compositional dissimilarity (β_{sim}) among polygons, performed a cluster analysis (UPGMA) and performed a simple permutation test for clusteredness[51] on 999 iterations to delineate the number of optimal bioregions described for each of those datasets. The number of delineated bioregions for each matrix is given in Supplementary Fig. 3a, which we then compared to the 5 bioregions delineated for non-native ant species only (309 species) and the 6 bioregions for all ant species at the global scale. A pool of at least 5000 species was needed to rebuild reliably the historical bioregions depicted by the all-ant species diversity (one sample t-test, $\mathrm{p-value}_{5000}$ = 0.1467, $\mathrm{p-value}_{10000}$ = 0.7976). All other random subsets (from 200 to 2000 included) lead to the splitting into a greater number of distinct assemblages (one sample t-test, p-value < 0.001), with the notable exception of the non-native species pool (309 species). In addition, we analyzed the spatial extent of geographic distributions of non-native ant species before human-mediated transport (*i.e.*, in their native range) and of native only ant species (Supplementary Fig. 3b). Based on this analysis, we found that although non-native ant species are a small subset of all ant diversity, their native ranges are representative of the historical bioregions were delineated for all ant diversity. On the other hand, a random selection of 300 ant species did not result in the same delineated bioregions as all ant species diversity. This is because most species have small ranges and drawing randomly 300 species is not sufficient to get a good estimation of global bioregions. The 309 native ranges of non-native ant species represented well the global bioregions of all ants because they are present in more polygons (Supplementary Fig. 3b, Wilcoxon test, p-value < 0.001) and more widespread (Supplementary Fig. 3c, Wilcoxon test, p-value < 0.001). As a consequence, they captured more of the spatial variation in all species turnover, compared to native only ant species.

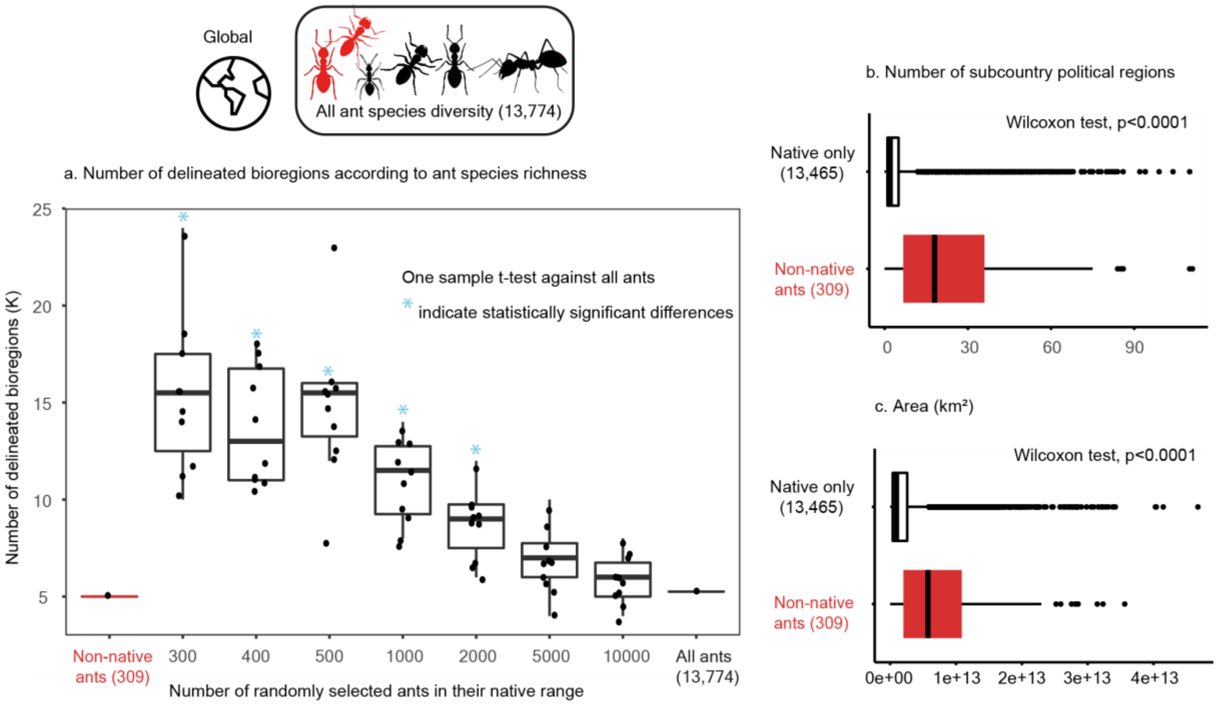

Supplementary Figure 3. (a) Number of delineated bioregions according to the size of the ant species pool (random subset of 300 to 10000 ants in their native range). (b) Number of polygons where non-native species are found in their native range (309 species) versus all native only species (13,465 species). (c) Native distribution (area in km^2) of non-native ant species versus all native only species.

Box plots represent data where the lower bound of lower whisker shows the minimum value of the data that is within 1.5 times the interquartile range under the 25th percentile, lower bound of box shows the lower quartile, center of box shows the median, upper bound of box shows the upper quartile, and upper bound of upper whisker shows the maximum value of the data that is within 1.5 times the interquartile range over the 75th percentile. Source data are provided as a Source Data file.

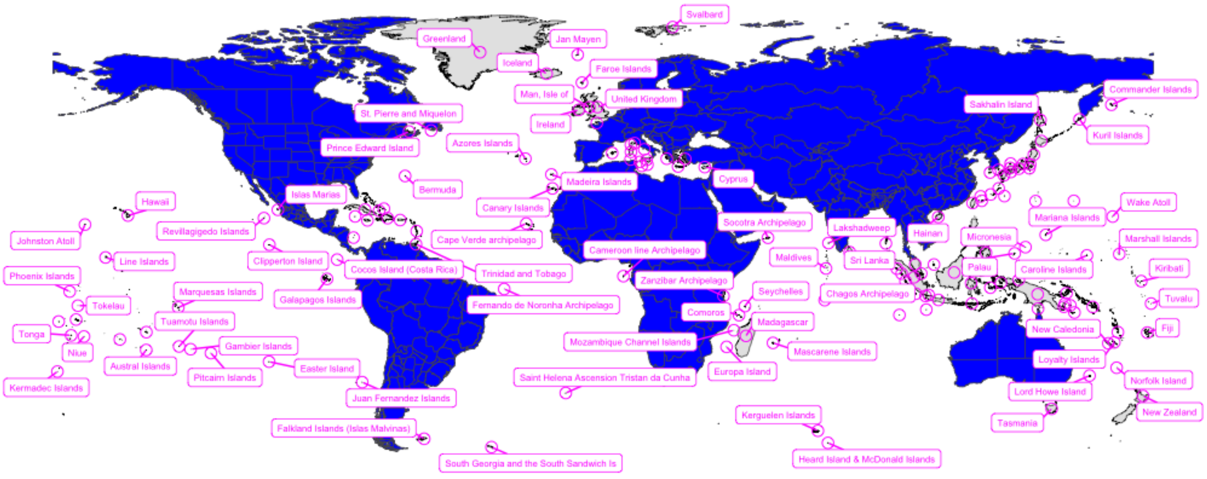

Supplementary Figure 4. Maps of polygons categorized by mainlands (blue) and islands (magenta).

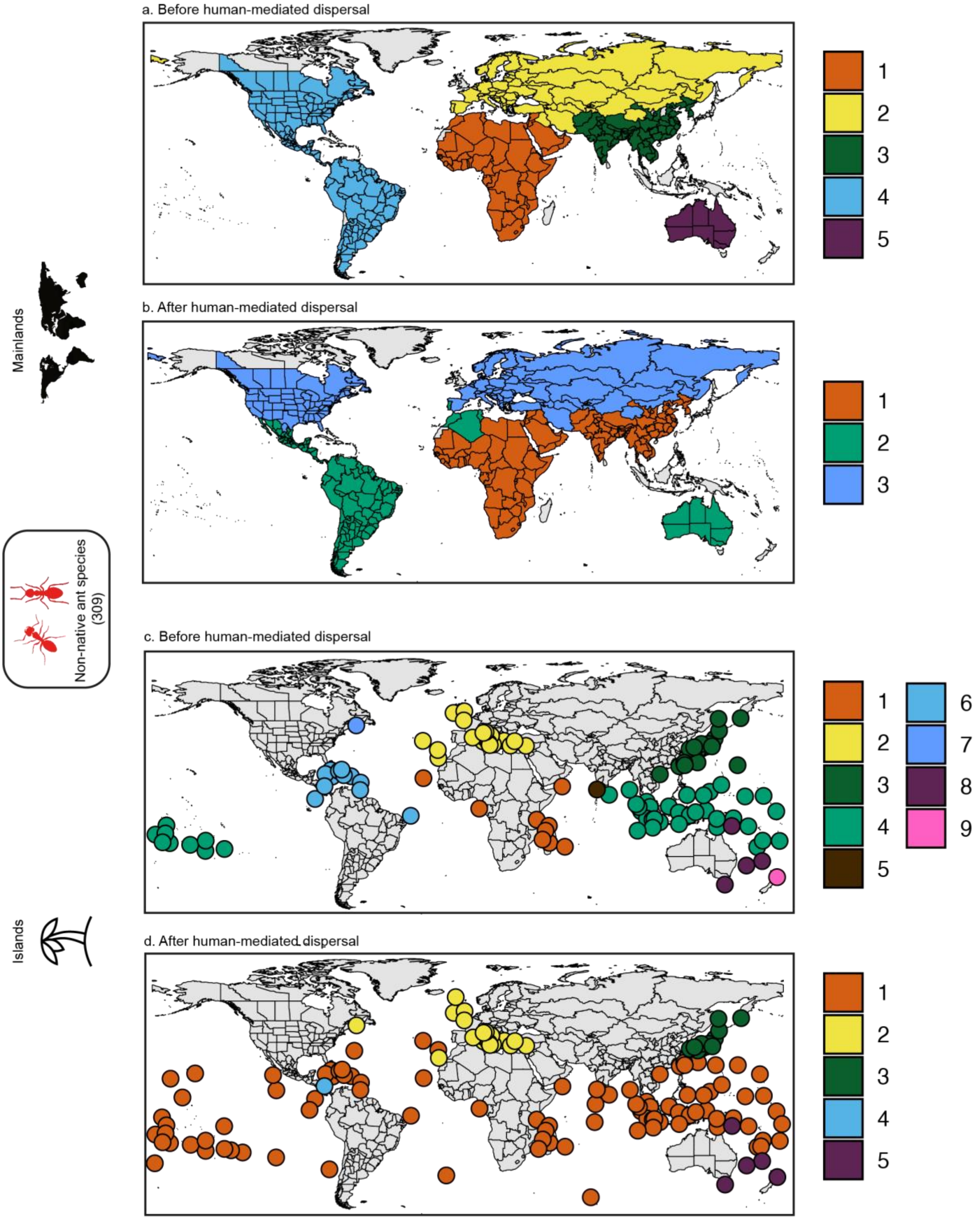

Supplementary Figure 5. Mainland (a-b) and island (c-d) biogeographic patterns of non-native ant species before and after human-mediated dispersal of non-native ant species.

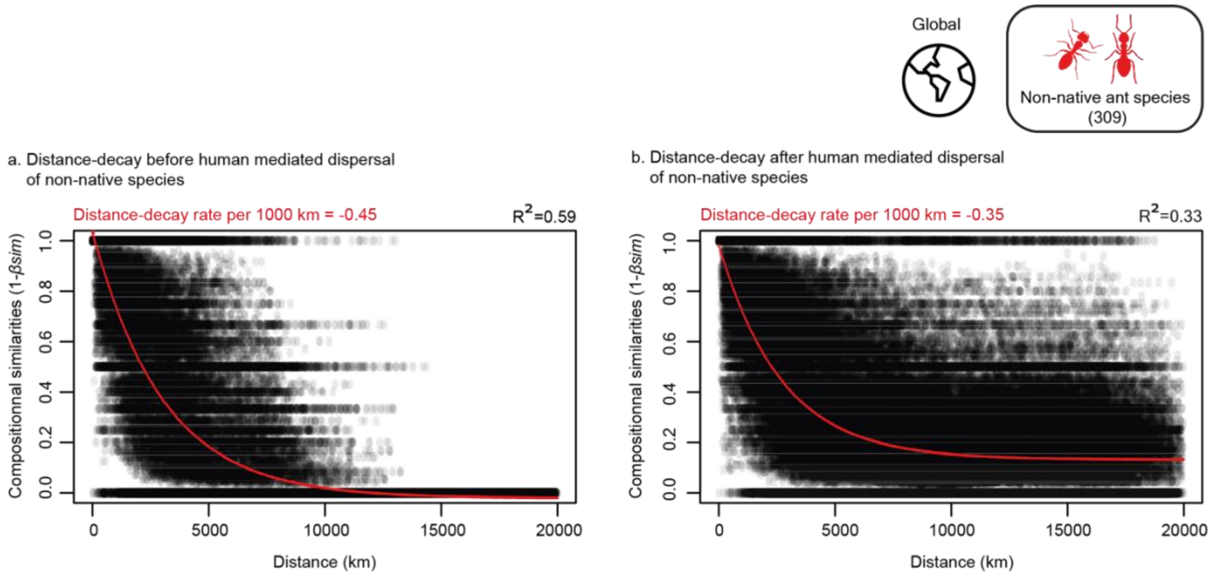

Supplementary Figure 6. Global distance-decay (a) before and (b) after human-mediated dispersal of non-native species for non-native ant species (309 species).

Distance-decay represents the relationship between compositionnal similarities (1-β_{sim}) of ant assemblages (for 309 non-native ant species) and geographical distances between the centroids of polygons before and after human-mediated dispersal of non-native ant species. Compositionnal similarities are fitted against distance with nls models. R-square of the models and distance-decay rate per 1000km are given above the graphs. Source data are provided as a Source Data file.
