# Supplementary material for: Non-native ants are breaking down biogeographic boundaries and homogenizing community assemblages
Source: Nat Commun. 2024 Mar 13;15:2266. doi: 10.1038/s41467-024-46359-9 (PMC10937723; doi:10.1038/s41467-024-46359-9)
Supplement: Supplementary file 3 — Reporting Summary [file 41467_2024_46359_MOESM3_ESM.pdf]

nature portfolio

Corresponding author(s): Aulus-Giacosa Lucie and Bertelsmeier Cleo

Last updated by author(s): 16.02.2023

Reporting Summary

Nature Portfolio wishes to improve the reproducibility of the work that we publish. This form provides structure for consistency and transparency in reporting. For further information on Nature Portfolio policies, see our Editorial Policies and the Editorial Policy Checklist.

Statistics

For all statistical analyses, confirm that the following items are present in the figure legend, table legend, main text, or Methods section.

n/a	Confirmed	
☐	☒	The exact sample size (*n*) for each experimental group/condition, given as a discrete number and unit of measurement
☒	☐	A statement on whether measurements were taken from distinct samples or whether the same sample was measured repeatedly
☐	☒	The statistical test(s) used AND whether they are one- or two-sided<br>*Only common tests should be described solely by name; describe more complex techniques in the Methods section.*
☐	☒	A description of all covariates tested
☐	☒	A description of any assumptions or corrections, such as tests of normality and adjustment for multiple comparisons
☐	☒	A full description of the statistical parameters including central tendency (e.g. means) or other basic estimates (e.g. regression coefficient) AND variation (e.g. standard deviation) or associated estimates of uncertainty (e.g. confidence intervals)
☐	☒	For null hypothesis testing, the test statistic (e.g. *F*, *t*, *r*) with confidence intervals, effect sizes, degrees of freedom and *P* value noted<br>*Give P values as exact values whenever suitable.*
☒	☐	For Bayesian analysis, information on the choice of priors and Markov chain Monte Carlo settings
☐	☒	For hierarchical and complex designs, identification of the appropriate level for tests and full reporting of outcomes
☒	☐	Estimates of effect sizes (e.g. Cohen's *d*, Pearson's *r*), indicating how they were calculated

Our web collection on statistics for biologists contains articles on many of the points above.

Software and code

Policy information about availability of computer code

Data collection	No software were used to collect the data.
Data analysis	Data processing and statistical analyses were undertaken in R (v.4.1.0; R Core Team, 2021) and RStudio (Version 2022.12.0+353).<br>We calculated pairwise matrices of compositional dissimilarity among polygons using the beta- diversity index (β_sim, vegan package, v2.5-7).<br>To identify biogeographic patterns, we performed a clustering analysis of the compositional dissimilarity matrices using an unweighted pair group method with arithmetic mean (UPGMA) (hclust, stats package, v4.2.2).<br>We used the Scheirer-Ray-Hare test (rcompanion package, v2.4.26), to test if average value of homogenization index was linked to their location on islands and/or within the tropics.<br>We calculated the location of each polygon centroid using sf package (v1.0-10).<br>We fitted the GLMMs using the Automatic Differentiation Model Builder GLMMADMB R package (v0.8.3.3).<br>We tested if the distance-decay relationship changed after human-mediated transport using non-linear least squares models of compositional similarity (nls, stats package, v4.2.2).<br>We used the Mantel statistic to test if the relationship between species assemblage similarity and geographic distance changed after human-mediated dispersal (mantel test, vegan package, v2.5-7).<br>Graphics and maps were produced using the ggplot2 (v3.4.1) and sf (v1.0-10) packages. Additional editing of figures were undertaken in Adobe Illustrator.<br>The full reproducible code is available in a Figshare repository at https://doi.org/10.6084/m9.figshare.22188208.v1.

For manuscripts utilizing custom algorithms or software that are central to the research but not yet described in published literature, software must be made available to editors and reviewers. We strongly encourage code deposition in a community repository (e.g. GitHub). See the Nature Portfolio guidelines for submitting code & software for further information.

Data

Policy information about availability of data

All manuscripts must include a data availability statement. This statement should provide the following information, where applicable:

- Accession codes, unique identifiers, or web links for publicly available datasets
- A description of any restrictions on data availability
- For clinical datasets or third party data, please ensure that the statement adheres to our policy

The raw data that support this study were sourced from the webmaps displayed on antmaps.org which is linked to the Global Ant Biodiversity Informatics (GABI) database and Wong et al. (2023). All processed data generated and analyzed in this study have been deposited in a Figshare repository accessible at https://doi.org/10.6084/m9.figshare.22188208.v1 . Source data are provided with this paper.

Research involving human participants, their data, or biological material

Policy information about studies with human participants or human data. See also policy information about sex, gender (identity/presentation), and sexual orientation and race, ethnicity and racism.

Reporting on sex and gender	N/A
Reporting on race, ethnicity, or other socially relevant groupings	N/A
Population characteristics	N/A
Recruitment	N/A
Ethics oversight	N/A

Note that full information on the approval of the study protocol must also be provided in the manuscript.

Field-specific reporting

Please select the one below that is the best fit for your research. If you are not sure, read the appropriate sections before making your selection.

☐ Life sciences ☐ Behavioural & social sciences ☒ Ecological, evolutionary & environmental sciences

For a reference copy of the document with all sections, see nature.com/documents/nr-reporting-summary-flat.pdf

Ecological, evolutionary & environmental sciences study design

All studies must disclose on these points even when the disclosure is negative.

Study description

The study aimed at delineating biogeographic reams for non-native ant species (309) and all ant species (13,758) both before and after human-mediated dispersal of non-native species. To do so we extracted native distribution from the Global Ant Biodiversity Informatics (GABI) database and current distribution of non-native species that established population outdoors from Wong et al. (2023). We produced matrices of presence/absence (species X polygons) at the global, mainlands and islands scale by categorising polygons. We then delineated bioregions for those 12 matrices, by using compositionnal dissimilarity measures then analysed using an unweighted pair group method with arithmetic mean (UPGMA).

To compare ant communities before and after human mediated dispersal, we additionnaly calculated an homogenization index equal to the difference in compositionnal dissimilarities. We used the Scheirer-Ray-Hare test, to test if homogenization was higher on islands versus on mainlands, or in tropical versus non-tropical locations.

To test if the number of non-native species that a region has received (i.e. non-native species arriving from elsewhere that have established in the focal region) or donated (species native to the region, with established non-native populations elsewhere) was linked to their location on islands and/or within the tropics, we used separate GLMMs.

Finally, we tested if the distance-decay relationship changed after human-mediated transport using nls models of compositional similarity as a function of distance between polygon centroids at the global scale. We then used the Mantel statistic to test if the relationship between species assemblage similarity and geographic distance changed after human-mediated dispersal.

Research sample

We extracted native distribution data of 13,774 ant species with valid species name based on AntCat.org from the Global Ant Biodiversity Informatics (GABI, 26.11.2020) database to delineate native biogeographic realms of all ant species. We additionnaly extracted the distribution in their native and in their non-native range of 309 non-native ant species (species that have succeeded in outdoor establishment outside of their native range) from Wong et al. (2023). We used the 309 non-native ant species to delineate both their native and current biogeographic regions. We used the current range distribution of all ant species (13,774 species in their native range + 309 species in their non-native range) to delineate current biogeographic realms of all ant species after the human-mediated dispersal of non-native ant species, to understand the ongoing biogeographic changes due to species introductions outside of their native range.

Sampling strategy	Species native ranges were sourced from the webmaps displayed on antmaps.org which is linked to the Global Ant Biodiversity Informatics (GABI) project ; the details of ant species distribution records are fully described in Guénard et al. (2017). For non-native species, we used the native and non-native ranges of 309 non-native ant species that have established outdoors, excluding non-native species that are only introduced indoors or intercepted at border controls, described in Wong et al. (2023). Sourcing our data in both public datasets ensure we did use the most up to date knowledge on ant species distribution.<br><br>From this dataset, we excluded species with unknown distributions as well as records which are listed as “dubious” or “needing verification”. Species distributions were formatted as presence/absence data at the geographical scale of the sub-country political regions (referred to as “bentities” in GABI, hereafter polygons), and absences were inferred as the lack of presence data. The polygons, described in Guénard et al., 2017, reflect human political delineations (e.g., country level; state), geology (e.g., mainlands, islands) and scientific knowledge (e.g., specific split of political entities). Out of a total of 546 polygons, ant species are found in 536. Our final dataset comprised the distribution of 13,774 ant species with valid species name based on AntCat.org and additional non-native ranges of 309 non-native ant species. The native records of non-native ant species were considered to correspond to the species’ ranges before human-mediated dispersal, while entire distribution including native and non-native ranges correspond to the species’ current ranges after human-mediated dispersal.
Data collection	The data for native ant species distribution were directly exported form the publicly available dataset GABI on the 26.11.2020 by Sebastien Ollier. The data for non-native ant distribtuion were extracted from the Supplementary material provided along with the paper of Wong et al. 2023 in February 2023 by Lucie Aulus-Giacosa. Raw data were stored at csv file.
Timing and spatial scale	The data for native ant species distribution were exported on the 26.11.2020 from GABI. The distribution of the 309 non-native ant species (species that have succeeded in outdoor establishment outside of their native range) were extracted from Wong et al. (2023) in February 2023. Both datasets were extracted at the global scale, with records of species distribution at the country or subcountry political regions scale (bentities defined in Guenard et al. 2017).
Data exclusions	We removed all records which are listed as “dubious” or “needing verification”, as well as records for non-native species that have not established population in the wild (indoor introduced).
Reproducibility	No experiments were undertaken. Data and codes are available to reproduce the full study in a Figshare repository at https://doi.org/10.6084/m9.figshare.22188208.v1.
Randomization	Native versus non-native ants.<br>We defined two groups of native and non-native ants based on the description of species distribution in their native range (GABI) and in their non-native range recorded in Wong et al. 2023.<br><br>Mainlands versus islands.<br>Among the 536 polygons where ant species are recorded, 384 were located on mainlands and 152 on islands. For this analysis, we defined an island as an area surrounded by water smaller than the smallest continent (with Greenland being therefore the biggest islands). To classify polygons as mainlands and islands, we used recent works on ant species distributions (Liu et al. 2023) and on invasive non-native species (Bodey et al. 2023) on islands. We did not considered Newfoundland as an island as most of its surface was comprised on mainlands.<br><br>Tropical versus non-tropical<br>A tropical versus non-tropical status was attributed to each polygon according to the location of each polygon centroid (sf package70,71, v1.0-10). Polygons for which the centroid was located between the two latitudinal parallels 23° far from the equator were considered as tropical.
Blinding	Data collection in our study did not require blinding, as we collected published data on ant species distribution.

Did the study involve field work? ☐ Yes ☒ No

Reporting for specific materials, systems and methods

We require information from authors about some types of materials, experimental systems and methods used in many studies. Here, indicate whether each material, system or method listed is relevant to your study. If you are not sure if a list item applies to your research, read the appropriate section before selecting a response.

Materials & experimental systems

n/a	Involved in the study
☒	☐ Antibodies
☒	☐ Eukaryotic cell lines
☒	☐ Palaeontology and archaeology
☒	☐ Animals and other organisms
☒	☐ Clinical data
☒	☐ Dual use research of concern
☒	☐ Plants

Methods

n/a	Involved in the study
☒	☐ ChIP-seq
☒	☐ Flow cytometry
☒	☐ MRI-based neuroimaging
